# Genetic Analysis of Kernel Traits in Maize-Teosinte Introgression Populations

**DOI:** 10.1534/g3.116.030155

**Published:** 2016-06-09

**Authors:** Zhengbin Liu, Arturo Garcia, Michael D. McMullen, Sherry A. Flint-Garcia

**Affiliations:** * Division of Plant Sciences, University of Missouri, Columbia, Missouri 65211; † U.S. Department of Agriculture, Agricultural Research Service, Columbia, Missouri 65211

**Keywords:** introgression population, kernel weight, kernel size and shape, quantitative trait loci (QTL), domestication, Multiparent Advanced Generation Inter-Cross (MAGIC), multiparental populations, MPP

## Abstract

Seed traits have been targeted by human selection during the domestication of crop species as a way to increase the caloric and nutritional content of food during the transition from hunter-gather to early farming societies. The primary seed trait under selection was likely seed size/weight as it is most directly related to overall grain yield. Additional seed traits involved in seed shape may have also contributed to larger grain. Maize (*Zea mays* ssp. *mays*) kernel weight has increased more than 10-fold in the 9000 years since domestication from its wild ancestor, teosinte (*Z. mays* ssp. *parviglumis*). In order to study how size and shape affect kernel weight, we analyzed kernel morphometric traits in a set of 10 maize-teosinte introgression populations using digital imaging software. We identified quantitative trait loci (QTL) for kernel area and length with moderate allelic effects that colocalize with kernel weight QTL. Several genomic regions with strong effects during maize domestication were detected, and a genetic framework for kernel traits was characterized by complex pleiotropic interactions. Our results both confirm prior reports of kernel domestication loci and identify previously uncharacterized QTL with a range of allelic effects, enabling future research into the genetic basis of these traits.

In cereal crops, seeds are the primary products for consumption, and seed size is one of the most important agronomic traits for yield. Seed size is also a vital component of evolutionary fitness in plants. It is believed that larger seeds accumulate sufficient nourishment for germination and have better tolerance to abiotic stresses, while smaller seeds are more efficient at dispersal and colonization (Westoby *et al.* 2002; Moles *et al.* 2005). Seed shape traits often govern market classes in some crops, such as long *vs.* short grain rice (Luo *et al.* 2004; Huang *et al.* 2013).

Researchers studying Old World archeological data (Fuller 2007) believe that larger grain size emerged before the loss of shattering during domestication, and that increased seed size compared to progenitors could be found in nearly every domesticated species. Grain shape was involved in a second step of crop improvement and diversification following domestication. Grain shape changes during domestication can also be dramatic (Abbo *et al.* 2014), although species would still be considered domesticated in the absence of grain shape changes, and the changes are often found in only a subset of domesticates (Gross and Olsen 2010). This is one possible reason why grain shape does not appear to have been a major component of the domestication syndrome in wheat (Gegas *et al.* 2010). Grain size and shape were both under strong selection during rice (*Oryza sativa*) domestication (Kovach *et al.* 2007).

Besides being important in domestication, kernel size and shape are crucial attributes for determining the market value of rice due to their close relationship to quality and yield. For example, studies indicated that larger and more spherical grains could increase the milling yield in wheat (Evers *et al.* 1990; Evers 1995) and are associated with cooking quality in rice (Luo *et al.* 2004; Huang *et al.* 2013). Thus, grain size and shape have attracted significant attention not only in cereal crop breeding programs, but also in genetic studies because of their contributions to yield, quality, and domestication. Understanding the genetic basis of these traits is crucial from both an evolutionary and applied perspective.

The mechanism by which grain crops determine their seed size and shape has remained largely unknown at the gene level and beyond. Most studies on seed size and shape have only identified QTL for grain size and shape in plants ranging from *Arabidopsis* to various crop plants (Liu *et al.* 2014; Zhang *et al.* 2015), which indicate that grain size and shape are quantitative traits. Recently, several genes/QTL controlling seed/fruit size and shape have been cloned in tomato and rice (Frary *et al.* 2000; Fan *et al.* 2006; Song *et al.* 2007; Shomura *et al.* 2008; Weng *et al.* 2008; Wang *et al.* 2012) or identified using mutant strategies in *Arabidopsis* (Garcia *et al.* 2005; Zhou *et al.* 2009). These studies revealed that ubiquitin pathway (Song *et al.* 2007; Li *et al.* 2010a), transcription factors (Garcia *et al.* 2005), G-protein signaling (Fan *et al.* 2006; Huang *et al.* 2009), hormone signaling (Jiang *et al.* 2013; Liu *et al.* 2015), and epigenetics (Song *et al.* 2015) are involved in controlling seed/fruit size and shape in plants.

Maize (*Zea mays* ssp. *mays*) was domesticated from its progenitor teosinte (*Z. mays* ssp. *parviglumis*) about 9000 years ago in southern Mexico (Matsuoka *et al.* 2002; Piperno *et al.* 2009). The process of domestication involved artificial selection that resulted in radically different plant, ear, and kernel morphologies between teosinte and maize (Beadle 1939; Doebley and Stec 1993). As is the case for all domesticated crop species, humans had selected for maize plants that were easier to harvest: reduced number of harvest units (ears in the case of maize), ears that did not naturally disperse their seeds by shattering, larger seed size, etc. These attributes collectively are referred to as the domestication syndrome (Harlan 1971; Hammer 1984). However, there are surprisingly few reports on the genetic control of kernel shape in maize (Liu *et al.* 2014), though QTL mapping has been applied to mine the genomic regions related to kernel shape in other crop plants. To date, studies on the genetics of maize kernel traits have focused mostly on kernel composition, such as oil, protein, and starch content (Goldman *et al.* 1993; Clark *et al.* 2006; Flint-Garcia *et al.* 2009; Cook *et al.* 2012), and seed weight (Beavis *et al.* 1994; Messmer *et al.* 2009; Liu *et al.* 2016). Thus, there is a need to investigate the genetics of kernel shape in maize.

In the current study, we used a population of teosinte NILs (Liu *et al.* 2016) derived from 10 *parviglumis* accessions in the B73 background to investigate the genetic architecture of kernel weight and shape traits. Grain samples from replicated trials were subjected to image analysis to estimate kernel shape parameters. We conducted joint linkage mapping for each trait separately and then on traits derived from a principal component (PC) analysis. We then focused on pleiotropic relationships between kernel weight and shape traits.

## Materials and Methods

### Genetic materials and field trials

Ten NIL populations were derived from geographically diverse *parviglumis* accessions by backcrossing into the B73 background as previously described (Liu *et al.* 2016). Briefly, *parviglumis* pollen was crossed onto B73 ears, and a single F1 plant was used to derive each population by backcrossing four times. BC_4_ plants were self-pollinated two generations prior to seed increase by sib-mating. One of the BC_4_ populations (Z031) was also inbred via doubled haploid technology (AgReliant Genetics, Westfield, IN).

Field trials were conducted at Genetics Farm (two replicates) and Bradford Farm (one replicate) locations near Columbia, MO in 2009 and at Aurora, NY (two replicates) in 2010. The experiments consisted of 694 lines from eight populations in 2009, and 858 lines from all 10 populations in 2010. Plants were planted in single row plots, where lines were randomized within population and populations were randomized within replicates. B73 was planted as a check entry at a rate of 5%.

### Phenotypic data collection

Ears were harvested at physiological maturity, and dried at 37° for 5 d, followed by long-term storage at low humidity at 4° for at least 1 yr. The weight of 50 kernels (Wt50k) was obtained from three ears per plot.

Kernel size and shape were obtained using a bulk of kernels from each row grown in Columbia in 2009 and Aurora in 2010, and analyzed with PAX-it (MIS, Inc.) digital imaging software. Specifically, about 50 kernels per ear were scanned on an HP Scanjet G4050 Photo Scanner (Hewlett-Packard). Kernel area, perimeter, length, width, and roundness were calculated by the software after images were captured. The ratio of length and width (L/W) and the factor form density (FFD) were calculated from the raw data. FFD describes the differences in grain density and is calculated as grain weight divided by the product of grain length and grain width in wheat (Giura and Saulescu 1996). Because we have accurate kernel area values, we modified the FFD in our study to mean kernel weight divided by kernel area, based on the differences in size and shape parameters between maize and wheat.

### Genotyping and genetic linkage map

The NILs were genotyped via a GoldenGate assay (Illumina, San Diego, CA) with 728 polymorphic SNPs from the NAM marker set (McMullen *et al.* 2009), with an average of 553 polymorphic SNPs per NIL population as previously described (Liu *et al.* 2016). Because the BC_4_ population structure does not permit genetic map construction, we used the NAM genetic map as a framework for marker order (Supplemental Material, Table S1).

The SNP genotypes were converted to 0, 1, and 2 to represent homozygous B73, heterozygous, and homozygous teosinte, respectively. Missing genotypic data (primarily monomorphic markers in specific populations) were imputed based on flanking marker data as previously described (Liu *et al.* 2016).

### Statistical analysis

Descriptive statistics analysis and normality tests on the phenotypic data were performed with SAS software (SAS 9.2, SAS Inc.). Heritability was calculated according to Holland *et al.* (2003) in SAS (SAS Institute Inc., Cary, NC). LSMeans across environments were calculated in SAS using PROC MIXED with entry, environment, environment by entry, and replicates within environment as random effects. LSMeans are provided in Table S2. PC analysis (PCA) was performed in SAS using the PROC PRINCOMP procedure. The correlation matrix-based method was used for PC extraction, and only PCs with eigenvalues equal to or greater than 1 were retained (Field 2009).

### Joint linkage mapping

LSMeans across environments were used for joint linkage QTL analysis by employing the PROC GLMSELECT in SAS as previously described (Buckler *et al.* 2009; Tian *et al.* 2011; Liu *et al.* 2016). Briefly, a stepwise regression model was used to fit the population term, and markers nested within population. Where an individual population lacked an introgression covering the test SNP, no test was conducted for that population. The significance level for entry in, and exit out, of the stepwise model was determined by 1000 permutations. PROC GLM was used to fit an additive model, where the allelic effects were considered fixed effects. Significant alleles were determined by a *t*-test comparison of the parental allele *vs.* the control B73 allele. QTL support intervals were calculated by adding a single flanking marker for the QTL at a step of 0.1 cM to the full model and testing the significance at the *P* = 0.05 level.

Pleiotropy between pairs of traits in the joint linkage analysis was evaluated for QTL with overlapping support intervals in SAS. Correlations between allelic effect estimates were used to detect the significant pleiotropic QTL. Pearson correlation coefficients were considered to be significant at raw *P* = 0.0083 after FDR correction (*P* = 0.05).

### Data availability

The authors state that all data necessary for confirming the conclusions presented in the article are represented fully within the article and its supplemental tables, or are available as datasets in Liu *et al.* 2016.

## Results

### QTL analysis of kernel weight

An in-depth QTL analysis of kernel weight has been described previously (Liu *et al.* 2016), based on the full dataset of eight populations evaluated in seven replications and an additional two populations evaluated in four replications. However, because we wanted to compare the genetic architecture of kernel weight and kernel shape in the same set of experimental materials, we reanalyzed kernel weights based on a reduced dataset of 10 populations from only the five replications where we also collected additional kernel size and shape data.

Using the reduced dataset, we identified five Wt50k QTL (Table S3), where three of the QTL were also identified exactly as in the analysis of the full dataset (Liu *et al.* 2016) and the other two QTL shifted chromosomal locations only slightly compared to the full dataset. These differences in QTL detection between the full (Liu *et al.* 2016) and current reduced datasets are likely due to the number of populations analyzed and the number of replications and environmental effects on the data included in the analysis.

### Phenotypic framework of kernel weight, size, and shape

We measured eight kernel-related traits in the ten introgression populations in a subset of locations/replicates (Table 1). Kernel size traits included area, perimeter, length, and width, while kernel shape traits included roundness and length/width ratio (L/W). We also estimated kernel density in a term called FFD by dividing Wt50k by kernel area. Broad-sense heritability for these traits ranged from 0.72 to 0.96, with length, perimeter, and area showing the highest heritability, and FFD had the lowest heritability (Table 1). The heritability of Wt50k varied slightly, from 0.87 (Liu *et al.* 2016) to 0.89 when analyzing the full and partial datasets, respectively.

**Table 1:** Descriptive statistics for the traits in this study

Traits	Locations (Reps)	No. Samples	Mean	SD	Minimum	Maximum	*H^2^*
Wt50k (g)	2 (5)	856	12.65	1.20	6.79	16.52	0.890
Area (mm^2^)	2 (5)	856	58.13	4.29	44.12	72.76	0.945
Width (mm)	2 (5)	856	6.51	0.29	5.61	7.57	0.876
Length (mm)	2 (5)	856	10.99	0.53	8.72	12.40	0.951
L/W	2 (5)	856	1.69	0.09	1.32	1.95	0.872
Roundness	2 (5)	856	81.14	1.83	76.38	89.56	0.877
Perimeter (mm)	2 (5)	856	32.10	1.37	27.00	36.34	0.956
FFD (mg/mm^2^)	2 (5)	856	4.35	0.27	2.52	5.45	0.721

Reps, replicates; No., number; Wt50k, weight of 50 kernels; L/W, length/width; FFD, factor form density. SD, standard deviation. H^2^, Broad-sense heritability.

Wt50k was highly positively correlated with all the original kernel size traits except roundness and L/W (Table 2). Roundness and L/W thus describe the kernel shape traits, but not seed weight. We found that roundness and L/W show significant but weak correlations with area, a critical kernel size trait, which indicates that kernel size and kernel shape in maize are independent of each other just as in wheat (Gegas *et al.* 2010).

**Table 2:** Pearson correlation coefficients among kernel weight and shape traits

	FFD	Area	Perimeter	Roundness	Width	Length	L/W
Wt50k	0.643**	0.779**	0.709**	0.034	0.674**	0.588**	−0.027
FFD		0.035	−0.003	0.249**	0.220**	−0.075*	−0.231**
Area			0.940**	−0.172**	0.704**	0.845**	0.165**
Perimeter				−0.389**	0.507**	0.925**	0.388**
Roundness					0.556**	−0.649**	−0.988**
Width						0.255**	−0.567**
Length							0.649**

* significant at *P* < 0.05; ** significant at *P* < 0.001. FFD, factor form density; L/W, length/width; Wt50k, weight of 50 kernels.

**Figure 1:**
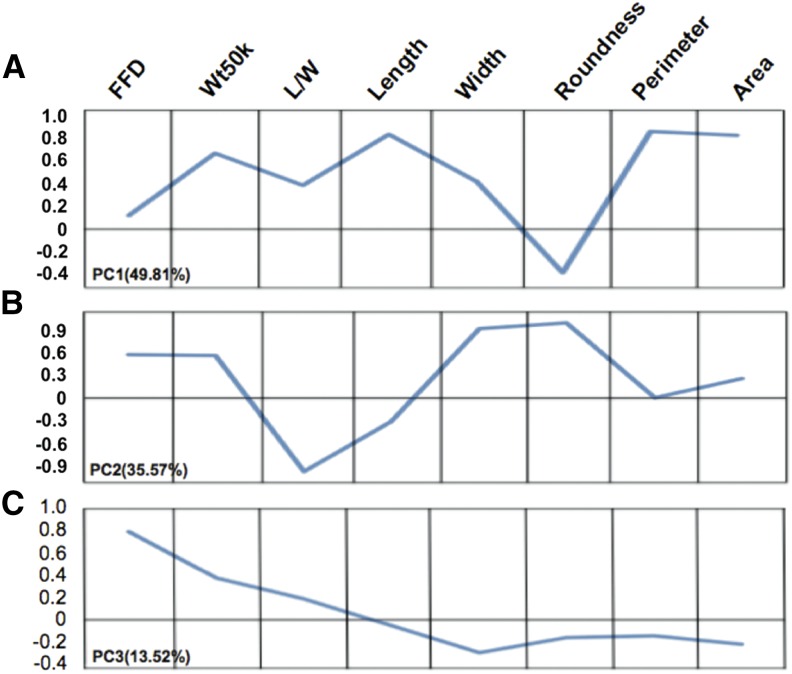
PCA of kernel weight, size, and shape traits. Wt50k variance is captured by all three PCs. (A) Area, perimeter, and length have large effects in PC1. (B) Roundness, width, and L/W have large effects in PC2. (C) PC3 describes variation in FFD. *y*-axis: component loading. Variance explained is annotated after each PC. FFD, factor form density; L/W, length/width; PC, principal component; PCA, PC analysis; Wt50k, weight of 50 kernels.

In order to simplify the relationships among kernel traits, PCA was performed on the kernel size and shape traits (Figure S1). Three significant PCs (PC1-3) with eigenvalues greater than 1 were extracted that together explained 98.9% of the variance (Figure S1, A and B). The PCs are linear combinations of the original kernel traits that are independent of each other, and represent different combinations of the traits based on their variable loadings (Figure 1): PC1 primarily represented variance in kernel size (area, perimeter, and length); PC2 mainly captured variance in kernel shape (roundness, L/W) and width; and PC3 was related to variance in kernel density (FFD). The first two PCs have almost equal (∼0.6) loadings for kernel weight, while the loading for PC3 is approximately 0.4. Because each of the PCs describes different combinations of kernel shape traits (*e.g.*, PC1 represents kernel size, PC2 represents roundness, etc.), each PC loading for kernel weight may reflect different components of kernel weight.

### Kernel trait QTL identified by joint linkage mapping

**Figure 2:**
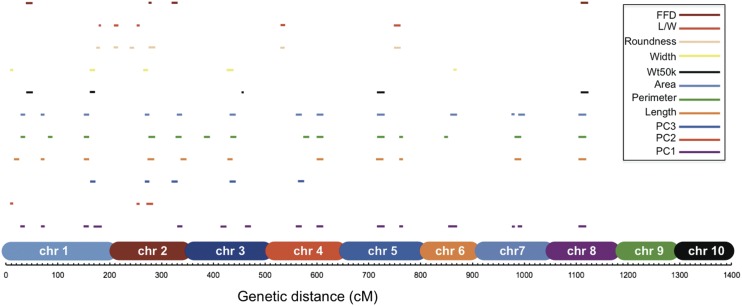
Distribution of QTL for kernel weight, size, and shape traits on genome. *x*-axis, the genetic distance (cM) of whole genome in NILs mapping population, and chromosomes 1–10 were labeled with different colors. The length of the QTL lines is proportional to the support interval length. Chr, chromosome; FFD, factor form density; L/W, length/width; NIL, introgression line; PC, principal component; QTL, quantitative trait loci; Wt50k, weight of 50 kernels.

We performed joint linkage QTL analysis on each of the original traits, as well as the PC traits, in order to identify the loci responsible for kernel trait differences between teosinte and maize. We identified 43 QTL for kernel size traits, 11 QTL for kernel shape traits, four QTL for FFD, and five QTL for Wt50k (Figure 2 and Table S3). The 63 QTL were distributed only on chromosomes 1–8, with no QTL on chromosomes 9 and 10. For the newly defined PC traits, PC1, PC2, and PC3, the total number of QTL detected were 15, 3, and 5, respectively (Figure 2 and Table S3). QTL for PCs collectively explained 62.51%, 21.70%, and 31.98% of the total variance, respectively, and were shared among seven to 10 populations (Table S4).

Several QTL were found in common between the derived PCs and kernel size, shape, and weight (Figure 2). For example, QTL for FFD, Wt50k, area, perimeter, length, and PC1 were identified on chromosomes 5 and 8. For the PC QTL, most had overlapping QTL for kernel size. For Wt50k, four of the five QTL had corresponding QTL for other kernel size, with the only exception of QTL for Wt50k on chromosome 3. These findings are consistent with the phenotypic framework analysis, where kernel weight was highly correlated with kernel size. Kernel weight is likely a composite trait controlled by various aspects of kernel shape, size, and density, and that any of these three aspects can be used to increase seed weight separately, as well as in combination. While PCA was able to simplify the relationships among the traits such that the components of kernel weight were split into the various PCs that are independent of each other, our data are not conclusive on the exact relationship between kernel weight and various size and shape traits.

**Figure 3:**
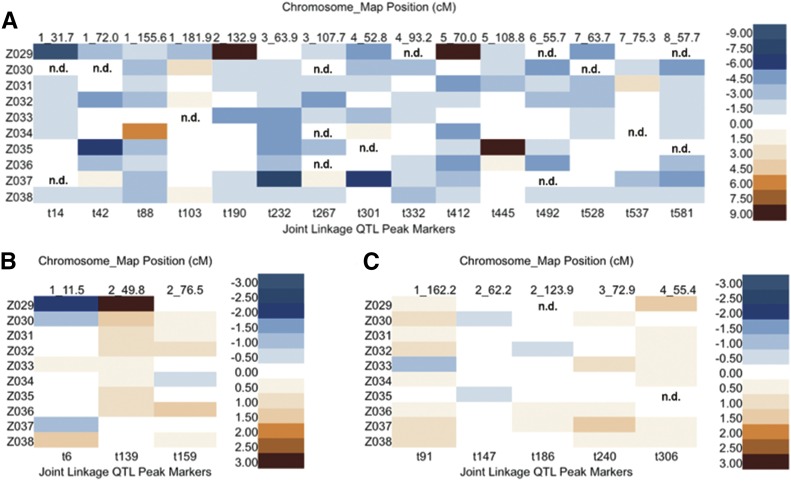
Heat map of additive effects for PCs QTL. The top horizontal axis lists the chromosome number and genetic map position for each QTL, while the bottom horizontal axis displays the QTL peak SNPs marker selected by linkage analysis. The vertical axis shows 10 NIL populations in order. Allelic affects are color coded with 2.00 increments for PC1 (A), and 0.50 increments for PC2 (B) and PC3 (C). n.d. indicates that allelic effects could not be determined, as there were no NILs carrying an introgression at the QTL. NIL, introgression line; PC, principal component; QTL, quantitative trait loci; SNP, single nucleotide polymorphism.

Additive allelic effects were estimated relative to B73 (Figure 3). While the biological meaning of PCs and their QTL effects may be difficult to interpret, their trait loadings can be used to represent a composite of correlated traits in a way that removes the interdependence among traits. Inherent to PCA, the first PC always captures the most variance, with subsequent PCs explaining decreasing amounts of variance. It is then not surprising that when we compared effects between PC1, PC2, and PC3, we found that PC1 effects had a much wider range (−8.08 − 24.82) than PC2 and PC3 (−2.22 − 4.95 and −1.33 − 1.82, respectively (Figure 3 and Table S4). Allelic series, or the detection of both negative and positive additive allele effects relative to the B73 allele, were identified for 60%, 67%, and 40% of the PC1, PC2, and PC3 QTL, respectively (Figure 3).

### Pleiotropic QTL affect kernel traits

**Figure 4:**
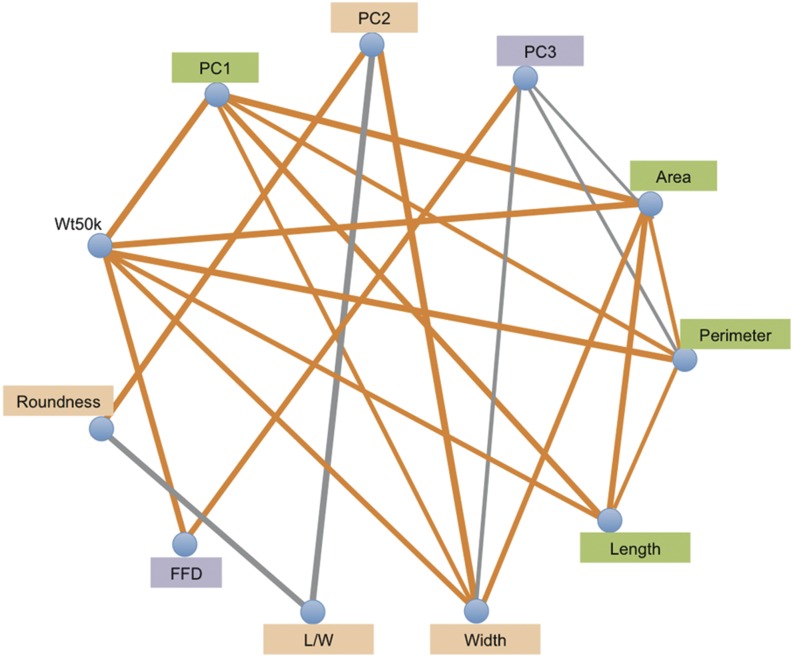
Pleiotropy for kernel traits in maize-teosinte NILs populations. Gray and orange lines between traits indicate negative and positive correlations between different QTL additive effects, respectively, with line width proportional to the degree of pleiotropy. FDR = 0.05 (raw *P*-value = 0.0083) was used as the significance threshold for effect correlation. FFD, factor form density; L/W, length/width; NILs carrying an introgression at the QTL. NIL, introgression line; PC, principal component; QTL, quantitative trait loci; Wt50k, weight of 50 kernels.

Maize-teosinte introgression populations provide substantial power to detect pleiotropy among overlapping QTL for multiple traits. Pleiotropy was assessed by correlating the allelic effects for overlapping QTL across the ten populations (Figure 4 and Table S5). If a QTL has large positive or negative effects for two traits in many of the same populations, the allelic effects at that locus will be significantly correlated and pleiotropy will be inferred. Positive pleiotropy was observed between kernel weight and kernel size traits (area, perimeter, and length) and was observed among the size traits themselves (Figure 4). Wt50k also had positive pleiotropy with FFD. In contrast to kernel size traits, the kernel shape traits (roundness and L/W) show negative pleiotropy with each other. This was expected as width is in the denominator of this ratio. Weak pleiotropy was observed between the kernel size and shape trait groups, which suggests that they have distinct genetic components. In addition, the PCA traits showed patterns of pleiotropy that were consistent with their observed phenotypic correlations with the kernel traits (Figure 4).

## Discussion

### Comparative genetic analysis revealed several candidate genes underlying maize kernel size and shape QTL

Seed size and shape are among the most important domestication syndrome traits for many plants, including rice, wheat, sorghum, and maize. Synteny and colinearity among land plants, especially among the grasses, allows us to analyze the genetic architecture of the same traits across species in a comparative manner. This comparative analysis can also help identify candidate genes underlying the QTL if candidates have been identified, cloned, and verified in other species.

In the current study, joint linkage mapping identified more than 60 QTL, including QTL for kernel area and length with moderate allelic effects, that colocalize with kernel weight QTL (Figure 2). Another strategy to map QTL is genome-wide association mapping (GWAS), which has higher mapping resolution, even to the single-gene level in species and populations with low linkage disequilibrium (Flint-Garcia *et al.* 2009). Unfortunately, the BC_4_ backcross population structure of the teosinte NILs is not a suitable platform for GWAS due to the limited recombination. The GWAS approach has been used in other species and maize populations to identify candidate genes for the same or similar traits. Interestingly, GWAS has poor power to identify rare alleles/variants even if they have a large effect. It was reported recently that several rare alleles are associated with grain size and yield in rice (Zhang *et al.* 2012; Song *et al.* 2015; Hu *et al.* 2015), and more are expected to be identified and verified in further studies. Hence, to date, linkage mapping has played a very important role in identifying rare alleles of intermediate to large effect, though GWAS methodologies are being developed to improve the identification of rare variants. In addition, transcriptional data are also helpful in the identification of candidate genes expressed in the appropriate tissues and time points (Sekhon *et al.* 2014). Here, we combine the findings of numerous studies which use mutant analysis, linkage mapping, GWAS, and transcriptomic analysis to understand and interpret our QTL results.

Using this comparative genetic analysis strategy, several candidate genes were identified for our kernel size and shape QTL, including known genes such as *ZmSH1 (*Lin *et al.* 2012*)*, *gln5* (Martin *et al.* 2006), *ZmGW2* (Li *et al.* 2010a), *and mn1* (Gupta *et al.* 2006), and unknown genes that have not been identified in maize but their orthologs were reported to relate to grain size and yield in other crop plants. For example, association analysis in sorghum association identified the gene *Sb04g015420* to be strongly associated with sorghum seed size (Zhang *et al.* 2015). The maize ortholog, *GRMZM2G00812*, has not been reported to associate with kernel size previously, but is potentially a candidate for our QTL on chromosome 5 (marker t412; Table S3).

FFD describes differences in grain density and has been defined as grain weight / (grain length × grain width) in wheat (Giura and Saulescu 1996). In our study, we modified the FFD trait to mean kernel weight divided by kernel area, based on differences in seed size and shape parameters between maize and wheat. The QTL on chromosome 2 controlling FFD has a strong candidate gene, *miniature 1* (*mn1*) (Gupta *et al.* 2006), under its peak. The maize gene *mn1* is the ortholog of *GIF1* in rice (Weng *et al.* 2008). *GIF1* encodes a cell wall invertase required for carbon partitioning during early grain fill. It is known that grain fill is an important trait that contributes greatly to grain weight, but the exact mechanism was unknown. Because this QTL was identified to control only FFD and not any other kernel size traits, we hypothesize that *mn1*-mediated grain filling might contribute to kernel weight through regulation of kernel density, not size, at least in maize.

### Mapping QTL with PCs identified some QTL that were not detected by univariate QTL analysis

To help understand the genetic control of multiple traits, we used PCA as our multivariate analysis method for related kernel traits in the study. PCA is widely used to decompose correlated variables into a smaller set of uncorrelated variables. The eigenvectors of the eigenvalues of the phenotypic covariance matrix, or the PCs, can be considered as new traits for QTL identification (Gilbert and Le Roy 2003; Topp *et al.* 2013). Mapping QTL with PCs can increase the statistical power to detect QTL by combining information across traits and removing the noise caused by trait correlations, and is used widely in both animal science (Gilbert and Le Roy 2003; Musani *et al.* 2006) and plant science (Choe and Rocheford 2012; Topp *et al.* 2013). The first three PCs together explained 98.9% of the variance in the original dataset (Figure S1, A and B), of which PC1 captured almost 50% of the variance (Figure 1A).

While PCA results in abstract PC values that have seemingly little biological meaning, these PCs are composite traits comprised of each of the original traits, as indicated by their different loadings for each trait (Figure 1, *y*-axis represents loadings), and can be used as phenotypes in QTL analysis (Topp *et al.* 2013). When comparing the QTL profiles for the individual kernel traits to PC-derived traits, we found that, in most cases, the PC-QTL analysis yielded similar results to QTL mapping with individual traits with patterns predicted by the PC loadings (Figure 1). However, PC-QTL analysis also identified additional chromosomal regions which were not detected by univariate analysis (*e.g.*, the two QTL on chromosome 3 for PC1; Figure 2 and Table S3). This identification of novel QTL is likely due to these QTL being associated with multiple traits.

### Genomic regions controlling domestication syndrome were identified

According to previous studies, a handful of genomic regions were believed to have strong effects on domestication traits (Doebley 2004). These regions were located on both arms of chromosome 1, the long arm of chromosome 3, and the short arms of chromosome 2, 4, and 5 (Doebley and Stec 1993; Doebley 2004). Our study not only identified QTL in these five regions, but also in additional regions located on chromosomes 2, 6, 7, and 8 (Figure 2 and Table S3). Many of our kernel phenotypes differed from these early studies, which included plant and ear morphology traits.

The genomic segment on long arm of chromosome 1 straddles the cloned domestication gene *teosinte branched 1* (*tb1*) (Doebley *et al.* 1995), a member of the TCP family of transcriptional regulators (Cubas *et al.* 1999). This region affected many kernel traits in our study, including kernel length, perimeter, area, width, and weight, and the new defined traits PC1 and PC3 (Table S3). The strongest of the kernel length QTL (marker t88; Table S3) has an alternate candidate gene, *YABBY*, under its peak. The *YABBY* gene is an important transcription factor involved in plant development and is an excellent candidate gene for maize kernel size, since it was reported to control the fruit size in tomato (Cong *et al.* 2008). In rice, *YABBY* was positionally cloned and named *OsSh1* (*Shattering 1*) because of its important role in seed shattering (Fukuta and Yagi 1998). Recently, *SbSh1* was map-based cloned in sorghum and it was revealed that *Sh1* genes were under parallel selection during maize, rice, and sorghum domestication (Lin *et al.* 2012). The dual role of YABBY in fruit size in tomato and shattering in grasses suggest that the *YABBY* gene could play a pleiotropic role in controlling seed size and shattering simultaneously in maize as well. While we acknowledge that QTL peaks are quite broad and can encompass hundreds of genes, it is nonetheless interesting to speculate about the possibility of parallel selection across species and multiple traits. Until this QTL is further characterized for each trait (*e.g.*, by map-based cloning), the mechanism of action for this transcription factor remains unknown.

The QTL on chromosomes 5 and 8 are also very important because of their pleiotropic effects. Both have strong effects on kernel weight, area, perimeter, and length (Figure 2), and the QTL on chromosome 5 may also affect KRN (Liu *et al.* 2016). The most likely reason for the pleiotropic effects of the QTL on chromosome 5 is its location near the centromere, where the low-recombination environment of the pericentromeric region causes clusters of genes to act as a single QTL. One strong candidate gene for the QTL on chromosome 5 is *ZmGW2*, one of the two maize orthologs of *GW2* which controls grain size and weight in rice. A QTL for kernel weight was mapped to the *ZmGW2* region, and *ZmGW2* was found to associate with kernel weight by altering kernel size in maize (Li *et al.* 2010a). The *GW2* gene, which encodes a RING-type E3 ubiquitin ligase, alters grain width and weight and with very little effect on grain length in rice (Song *et al.* 2007). Interestingly, it seems that *GW2* affects kernel weight differently in maize than in rice, as our QTL affects kernel length but not width as is the case in rice. *GS3*, a gene that controls grain length and weight in rice (Fan *et al.* 2006), was also found to operate differently in maize (Li *et al.* 2010b) and wheat (Gegas *et al.* 2010).

### Pleiotropy analysis revealed the potential to improve yield and kernel composition in maize

One of the greatest challenges in breeding high-quality varieties is the strong phenotypic correlation among kernel quality traits (Simmonds 1995; Ge *et al.* 2005). For the crop breeding community, it has been a long standing problem that they have been able to improve either yield or quality, but almost never improve both simultaneously. Most improvements in quality are usually accompanied by a reduction in yield because of this close association. Our study showed that this problem also exists for kernel size and shape as these traits are significantly correlated both phenotypically and genetically (Figure 4 and Table 2). Because kernel size and shape were believed to relate to kernel yield and quality, respectively, in rice and wheat. Understanding the genetic basis of kernel size and shape, as well as any possible genetic constraints preventing the maximization of both traits, is especially crucial from the applied perspective. After the initial genetic analysis of kernel size and shape, we conducted pleiotropy analysis for the same kernel size and shape traits, and identified some alleles that alter one trait without affecting the others. For example, no interactions were found between kernel size traits (area, perimeter, and length) and the kernel shape traits of L/W and roundness. This suggests that kernel size and shape are different traits under substantially independent genetic control, could have evolved independently, and could be manipulated separately in breeding programs. This study has shown the close relationship between kernel size and kernel weight in maize. However, it is still not clear whether there is any relationship between kernel shape and kernel composition in maize. Meta-analysis to link kernel size and shape to kernel composition is a potential way to elucidate this relationship in the future. Our analysis of pleiotropy provides insight for breeding and biotechnology strategies to alter maize kernel weight, which may also have applications in other crop plants.

## Supplementary Material

Supplemental Material
