# Supplementary material for: Genetic Analysis of Kernel Traits in Maize-Teosinte Introgression Populations
Source: G3 (Bethesda). 2016 Jun 9;6(8):2523–30. doi: 10.1534/g3.116.030155 (PMC4978905; doi:10.1534/g3.116.030155)
Supplement: Supplemental Material [file supp_6_8_2523__index.html]

Genetic Analysis of Kernel Traits in Maize-Teosinte Introgression Populations — Supplemental Material 

# Genetic Analysis of Kernel Traits in Maize-Teosinte Introgression Populations

## Supplemental Material for Liu *et al.*, 2016

**Files in this Data Supplement:**

- Figure S1 - Principal component analysis of kernel weight and shape traits. (.pdf, 205 KB)
- Table S1 - Genetic map and imputed genotypes of the BC4S2/DH NILs. (.xlsx, 44 KB)
- Table S2 - LSMeans for kernel traits. (.xlsx, 139 KB)
- Table S3 - Table for all QTLs. (.xlsx, 17 KB)
- Table S4 - Allele effect for all QTLs. (.xlsx, 97 KB)
- Table S5 - Pleiotropy in joint linkage analysis. (.xlsx, 15 KB)
