# Supplementary material for: Genetic Analysis of Kernel Traits in Maize-Teosinte Introgression Populations
Source: G3 (Bethesda). 2016 Jun 9;6(8):2523–30. doi: 10.1534/g3.116.030155 (PMC4978905; doi:10.1534/g3.116.030155)
Supplement: Supplemental Material [file supp_g3.116.030155_FigureS1.pdf]

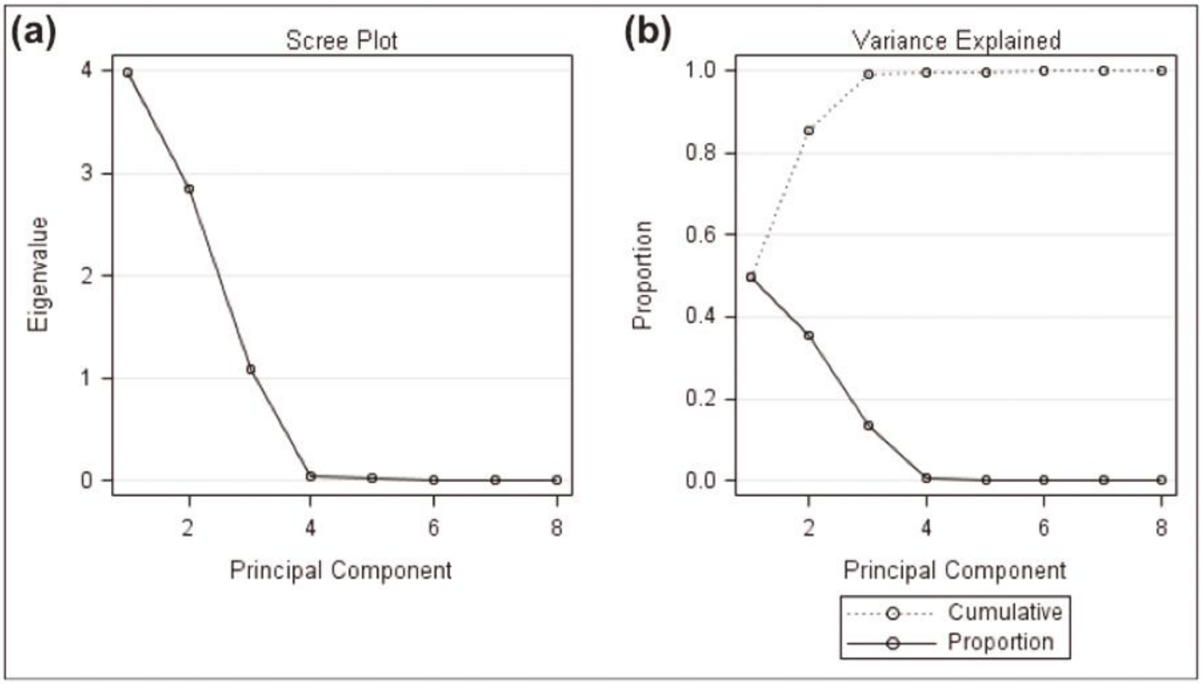

Figure S1. Principal component analysis of kernel weight and shape traits. (a) Eigenvalues of principal components (PCs). PCs with eigenvalue greater than 1 were retained. (b) Variance explained by different PCs. Dashed line represents the cumulative variance, while solid line represents variance explained by single PC
